# Activity of Mannose-Binding Lectin on Bacterial-Infected Chickens—A Review

**DOI:** 10.3390/ani11030787

**Published:** 2021-03-12

**Authors:** Peter A. Idowu, Adeola P. Idowu, Oliver T. Zishiri, Takalani J. Mpofu, Edwin J. A. Veldhuizen, Khathutshelo A. Nephawe, Bohani Mtileni

**Affiliations:** 1 Department of Animal Sciences, Tshwane University of Technology, Private Bag X680, Pretoria 0001, South Africa; MpofuTJ@tut.ac.za (T.J.M.); NephaweKA@tut.ac.za (K.A.N.); MtileniB@tut.ac.za (B.M.); 2 Department of Animal Science, North West University, Private Bag X2046, Mmabatho 2735, South Africa; patolagift@gmail.com; 3 Discipline of Genetics, School of Life Sciences, University of KwaZulu-Natal, Private Bag X54001, Durban 4000, South Africa; Zishiri@ukzn.ac.za; 4 Department of Biomolecular Health Sciences, Division Infectious Diseases and Immunology, Section of Immunology, Faculty of Veterinary Medicine, Utrecht University, 3584 CS Utrecht, The Netherlands; E.J.A.Veldhuizen@uu.nl

**Keywords:** chickens, use of antibiotics, innate immunity, lectin pathway, complement system, mannose-binding lectin quantification method

## Abstract

**Simple Summary:**

In the quest to combat bacterial-related diseases in chickens, different methods, of which some are less economical and less effective on the long-term, have been adapted. However, chickens possess mannose-binding lectin (MBL) which could be vital in managing pathogenic bacteria in chickens. MBL is one of the soluble proteins secreted by the chicken’s innate immune system which can be activated when chickens are exposed to chicken-related diseases. This review explains how mannose-binding lectin activation can help in fighting bacterial pathogens in chickens. This knowledge is believed to reduce incessant use of antibiotics and to assist in developing a profitable breeding program with less or no adverse effect on the chicken, human and the environment.

**Abstract:**

In recent years, diseases caused by pathogenic bacteria have profoundly impacted chicken production by causing economic loss in chicken products and by-product revenues. MBL (mannose-binding lectin) is part of the innate immune system (IIS), which is the host’s first line defense against pathogens. The IIS functions centrally by identifying pathogen-specific microorganism-associated molecular patterns (MAMPs) with the help of pattern recognition receptors (PRRs). Studies have classified mannose-binding lectin (MBL) as one of the PRR molecules which belong to the C-type lectin family. The protective role of MBL lies in its ability to activate the complement system via the lectin pathway and there seems to be a direct link between the chicken’s health status and the MBL concentration in the serum. Several methods have been used to detect the presence, the level and the structure of MBL in chickens such as Enzyme-linked immunosorbent assay (ELISA), Polymerase Chain Reaction (PCR) among others. The concentration of MBL in the chicken ranges from 0.4 to 35 µg/mL and can be at peak levels at three to nine days at entry of pathogens. The variations observed are known to depend on the bacterial strains, breed and age of the chicken and possibly the feed manipulation strategies. However, when chicken MBL (cMBL) becomes deficient, it can result in malfunctioning of the innate immune system, which can predispose chickens to diseases. This article aimed to discuss the importance and components of mannose-binding lectin (MBL) in chickens, its mode of actions, and the different methods used to detect MBL. Therefore, more studies are recommended to explore the causes for low and high cMBL production in chicken breeds and the possible effect of feed manipulation strategies in enhancing cMBL production.

## 1. Introduction

Chicken production plays a vital role in meeting the increasing demand for chicken products (such as meat and eggs) by using adequate management practices and structured breeding programs [1]. Despite the improvement in poultry management practices, bacterial disease is still a big threat to the chicken production industry [1,2,3]. For instance, countries like United States of America, China, Southern Europe and Portugal accounted for up to 50% loss in poultry product due to bacterial infections [2,3]. This problem has led to the incessant use of antibiotics due to their ability to improve feed efficiency, weight and poultry health. However, the use of antibiotics has been found to cause the emergence of antimicrobial resistant genes in chickens [3]. These antimicrobial resistant genes are then transferred between bacteria in the environment and into human pathogens [2,3] thereby causing serious health issues. For this reason, antibiotic use for growth promotion has been banned in some countries. Froebel et al. [4] stated that some government policies such as the United States Veterinary Feed Directive and the European Union are discouraging use of antibiotic growth promoters. There is, therefore, an urgent need to find alternative measures to manage bacterial diseases without compromising the health of chickens, humans or the environment.

The defense mechanism of chickens against pathogenic diseases entails the activity of both the innate and adaptive immune systems. Bacterial diseases are combated by an innate immune system (IIS) before the intervention of the adaptive immune system [5,6,7]. The IIS response is the host’s first line defense mechanism against any microbial infection; it functions by fighting pathogens at the point of entry via both innate immune cells and innate immune proteins [8,9]. On the other hand, the adaptive immune system ensures protection against subsequent infections by the action of both T and B lymphocytes [10] with activity against specific pathogens. This study seeks to review the innate immune system and, specifically, the activity of an important innate immune effector protein, mannose-binding lectin, in combating bacterial disease in chickens.

## 2. Brief Overview of the Chicken Innate Immune System

The IIS response is the host’s first line defense mechanism against microbial infection. For example, it functions by fighting pathogens at the point of entry via both innate immune cells and secreted soluble effector proteins [9]. The main innate immune cells include phagocytes such as macrophages and heterophils (comparable to the mammalian neutrophils), but also epithelial cells play an important role in the immediate response towards invading pathogens. The specific innate immune proteins are multiple and include complement system proteins, cytokines and chemokines (often secreted by the innate immune cells) and secreted soluble effector proteins, such as collectins and antimicrobial peptides, that can also kill or neutralize microbes [9]. 

An important first step in the innate immune response is recognition of the pathogen. There are specific structures on the microbe called pathogen-associated molecular patterns (PAMPs) which are recognized by membrane bound, or soluble pattern recognition receptors (PRRs) of the host. Larsen et al. [11] and Faghfouri et al. [12], both showed that toll-like receptors (TLRs), retinoic acid-inducible gene-I-like receptors (RLRs) and C-type lectin receptors (CLRs) are an important group of PRRs that can recognize PAMPs from microbial DNA/RNA to protein and membrane components of microbes. When PAMPs bind to PRRs, it leads to a signaling cascade which in turn increases cytokine and chemokine production; these molecules can activate immune cells. In some cases, through chemotaxis, the numbers of immune cells are increased at the site of infection. Signaling can also increase the production and secretion of antimicrobial compounds, such as defensins or other antimicrobial proteins that can neutralize the pathogens [13,14].

## 3. Mannose-Binding Lectin

Mannose-binding lectin (MBL) is also considered a member of the PRR family. In contrast to other PRRs such as TLRs, it is not membrane bound but a soluble-type protein which can also be considered as an effector molecule. Binding of MBL to PAMPs on the pathogen cell wall can initiate the lectin pathway of complement activation, which further neutralizes pathogens by aggregating them, (denying attachment of bacteria to epithelial cells of host). MBL can also bind apoptotic cells via ligation (joining of two DNA strands by a phosphate ester linkage) on the phagocyte membrane by macrophages [15]. The removal of apoptotic cells is necessary for organogenesis, tissue maintenance and proper activity of the immune system. MBL is also regarded as an acute-phase protein (proteins which respond to inflammatory signals by increasing its plasma concentration by 25% or more). These proteins participate in host defense and adaptation and also act as transport proteins with antioxidant activity [15,16]. Mannose-binding lectin is an innate host defense molecule, with great affinity to initiate the lectin pathway of complement via attached mannose-binding lectin-associated serine protease (MASP-2) [17,18,19]. The MBL structure provides a wide array of defenses against pathogens’ physiochemical activities as mentioned in the study of Takahashi [7]. Several authors have indeed discussed the impact of MBL on bacterial infection in chickens [15,16,17,18]. This paper seeks to review the structure, mode of action, and MBL activity on bacterial diseases in chickens, as well as the detection and quantification methods used for determining MBL concentrations and possible factors that affect MBL levels in chickens. 

### 3.1. MBL Structure

**Figure 1:**
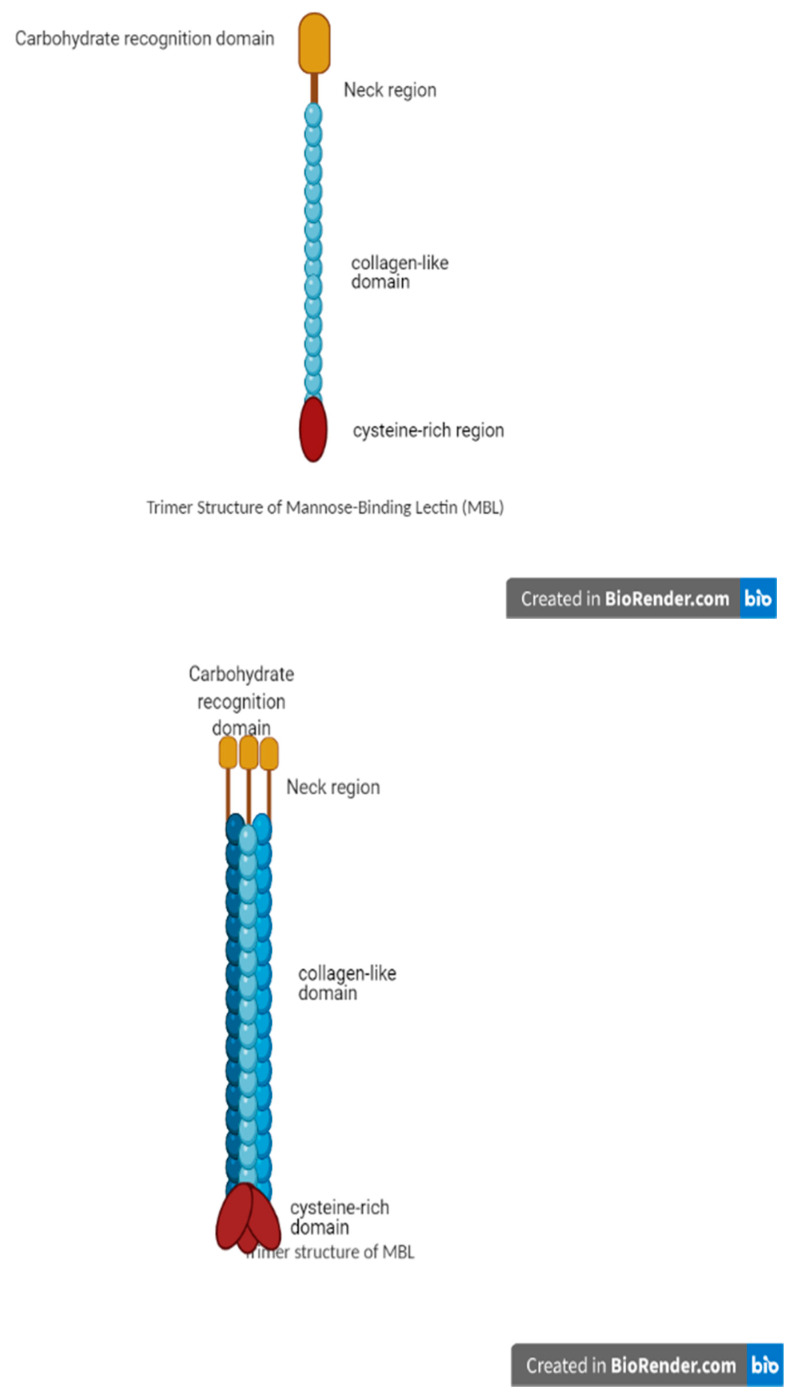
Set of domain of the collagenous MBL (mannose-binding lectin).

**Figure 2:**
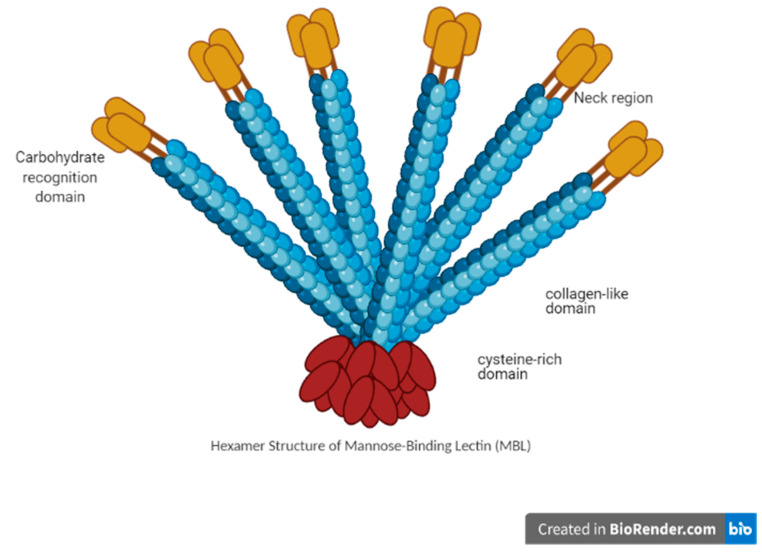
The multimeric structure of mannose-binding lectin (MBL).

MBL belongs to the C-type lectin family, and more specifically, to the family of collectins [5,7,8,9,11]. MBL are multimeric proteins comprising of many subunits where an individual subunit possesses three identical polypeptide chains (Figure 1). In the plasma of chicken, MBL’s size ranges from single subunit to oligomers of six or more subunits (Figure 2). The MBL structure has a short N-terminal domain and a large collagen domain, which is subsequentially linked to the lectin domain through its neck domain (Figure 1). 

The neck domain initiates trimerization of the monomers which is stabilized by the intertwined collagen of MBL [9,20]. The trimer is considered the smallest active form of MBL (structurally, the smallest form of MBL) [20]. The N-terminal domains can covalently crosslink the MBL trimers via the cysteine residues, to higher oligomeric structures (Figure 2). The International Union of Immunological Societies (IUIS) had not defined a specific oligomeric structure for MBL, and, in nature, several forms of MBL are present across animal species [9,19,20,21]. Laursen et al. [22] observed that purified chicken MBL has a similar structure as mammalian MBL [9,19,20,21].

### 3.2. MBL Mode of Action

There is no clear mechanism of action between serum level of MBL and disease parameters in reducing infections. What is clear is that binding of MBL via its lectin domain to glyco-conjugates on the bacterial cell membrane is the first step towards neutralization of pathogens [22,23]. The MBL later induces different biological activities such as phagocytosis, protein synthesis and transmission, modulation of inflammation, cell to cell interaction, signal transduction and cytokine production control at protein and mRNA levels [18,24]. The order of binding affinity of MBL on monosaccharides is as follows: ManNAc > l-fucose > mannose > GlcNAc [17,25].

For chicken MBL, the evidence is scarce whether agglutination of bacteria is an important factor in vivo in neutralizing bacteria. However, in vitro MBL is indeed capable to agglutinate *Salmonella Typhimurium* [18]. In addition, the study of Ulrich-Lynge et al. [18] observed that purified cMBL was able to bind all the serotypes of S. enterica except for the C1 serotype, while also viruses (although outside of the scope of this review) were agglutinated by MBL. Besides direct neutralization, binding of MBL to pathogens can lead to opsonization and increased phagocytosis of bacteria [8]. This has been well established for mammalian MBL, but only few studies have determined this MBL activity in chickens. It was clearly shown that binding of MBL led to an increased phagocytosis of Salmonella [18,19]. The third potential outcome of MBL binding to glycoconjugates on bacteria membranes is activation of the complement system.

### 3.3. MBL Ligand Binding

MBL is a significant member of the PRR family with ability to recognize PAMPs ranging from microbial DNA/RNA to protein and membrane components of microbes [19,20]. This binding requires a calcium ion (characteristic for C-type lectins) which links the hydroxyl side chains of the sugar residue to specific amino acids in the binding pocket of the lectin domain [26,27]. The conserved cysteines in the lectin domain ensure stability of the double loop structure of MBL. In general, the specificity for specific glycans is determined by specific amino acids. The characteristic amino acid motif ‘EPN’ in the binding pocket of C-type lectins results in a preference for mannose while ‘QPD’ leads to galactose-type lectins [27,28]. The study of Ulrich-Lynge et al [18] and Zhang et al [19] observed that MBL with mainly an EPN motif binds to high mannose glycan surface of microbes, while limited binding was observed in the QPD motif of galactose-rich region of microbes [18,19]. 

Nevertheless, the binding capacity of MBL in general is not limited to mannose or galactose alone but also sugars having 3- and 4-OH groups in the equatorial region of its sugar ring [27,28]. Such sugars are glucose, N-acetylmannosamine (ManNAc), N-acetylglucosamine (GlcNAc) and L-fucose. This kind of binding specificity is also called monosaccharide-binding specificity (MBS) [27]. The MBL identifies simple sugar and/or its by-products, and its reliability depends on the presence of a monosaccharide-binding pocket in the carbohydrate-recognition domain (CRD) [9]. Besides binding glycoconjugates, MBL also has affinity and capacity to bind nucleic acids, phospholipids and non-glycosylated proteins, but the effectiveness of this activity is less understood. An extensive description of all interactions between the glycoconjugates and the lectin domain which determines MBLs’ specificity for specific ligands is described in the study of Veldhuizen et al. [28].

In an attempt to test cMBL binding affinity to bacteria, studies have used flow cytometry [18,29]. This method uses dual trigger tools such as flourescent and forward scatter to detect cMBL binding affinity on bacteria, irrespective of the sample size [18,30]. In conclusion, MBL can bind to carbohydrate PAMPs on Gram-negative, Gram-positive and yeast, as well as some viruses and parasites.

## 4. Complement System 

**Figure 3:**
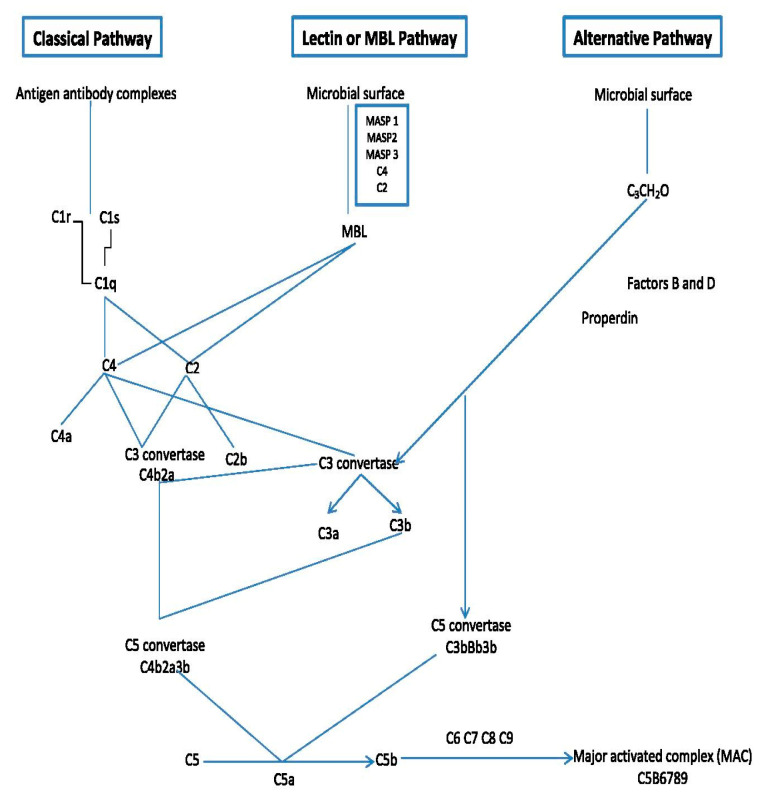
Outline of the major components and mode of action of the complement system.

The complement system (CS) plays a very important role in defending the host against diseases and inflammation. It is known that the CS consists of more than 30 soluble or membrane proteins that upon activation interact (the complement cascade), eventually leading to formation of an antibacterial complex of proteins and formation of several signaling molecules that stimulate further immune responses (Figure 3) [6,31]. 

The order of events of the complement cascade is as follows: Firstly, the complement cascade is activated via one of three different but eventually merging pathways: the lectin pathway, the classical pathway and the alternative pathway [6,31,32,33,34,35]. These pathways bring forth the formation of convertase enzymes which form several chemokine and other important signaling factors including the complement factor (C5b). 

All these pathways converge at C3, which is the most abundant complement protein found in the blood. At the point of converging, there is a formation of activation products such as C3a, C3b, C5a and the MAC or C5b-9 (Figure 3). The membrane attack complex (MAC) is a protein complex that can bind and lyse Gram-negative bacteria. It becomes triggered within a few minutes upon entry of Gram-negative bacteria in the host cells [31,36]. More details on how the complement system functions can be further seen in excellent reviews of Sarma and Ward, [6] and Heesterbeek et al. [37]. For this review, we will only be discussing the lectin pathway, which is the pathway activated by MBL.

### The Lectin Pathway of Complement Activation

When MBL binds a sugar on the microbial wall, the complement cascade becomes activated. The activation of complement in an MBL-dependent way was first described by Ikeda et al. [38]. MBL has the capacity to activate complement system through proteolytic enzymes called MBL-associated serine protease (MASPs) [8]. Another type of protein termed ficolin can also activate complement via the lectin pathway. Ficolin recognizes the outer layer structure of pathogens [32]. Both ficolin and MBL are present in the serum as complexes with MBL-associated proteins (MASPs). The MASPs are known to have evolutionary pedigrees, disposition, and roles similar to the C1 complex found in the classical pathway [39]. It is important to know that of all MASPs, only MASP-2 is vital for the activation of the lectin pathway. In fact, there are five basically linked MASPs: MASP-1, MASP-2, MASP-3, MAp19 (truncated MASP-2) and MAp14. The MASP-1, MASP-2 and MASP-3 are called proteolytic enzymes while MAp19 and MAp14 are non-enzymatic factors [40,41,42,43,44,45]. MASP-1 and MASP-2 are triggered to knit complement components C4 and C2. The C4 forms C4a and C4b while C2 forms C2a and C2b. The C4b and C2a forms C4bC2a which is also known as C3 convertase [46]. C3 convertase join C3 to C3b, while the C3b knits C4bC2a to form C5 convertase (C4bC2aC3b). The C5 convertase then splits C5 to C5a and C5b; this splitting begins the activity of the membrane attack complex (MAC). The C5b, therefore, incorporates C6, C7, C8, and C9 to produce a fully functional MAC (Figure 3), which results in the eradication of pathogen cells [8,39,47]. It is important to realize that information on the activity of the complement system is based on the mammalian system. For chickens, the information on the actual functionality of complement is scarce. However, (almost) all components of the complement cascade seem to be present in chickens with relatively high homology to their mammalian counterparts [31,34]. For example, based on genomic analysis, cMBL was observed to be closely related to human and/or rat MBL. Additionally, chicken has two ficolin genes of ficolin 1 (FCN 1) and ficolin 2 (FCN 2), again, quite homologous to mammalian ficolin [40]. These observations indicate strongly that chickens have a working complement cascade with a similar activating role for MBL, as seen in mammals.

## 5. Applicable Methods for Detecting and Quantifying MBL 

Several methods have been utilized to determine and quantify levels of cMBL. These methods vary in several aspects such as cost, precision, quantification or qualification strengths, as well as differences in precision and reproducibility. An overview of these characteristics is shown in Table 1 and Table 2. In this section of the manuscript, the different methods previously utilized by researchers and the limitations upon utilization of certain methods will be discussed.

### 5.1. Chromatography 

The affinity of MBL to bind carbohydrates can be utilized in chromatography. This method has the capacity to detect different types of lectins. The choice of the appropriate matrix and ligand are some of the challenges faced in using this method. In some cases, additional separation techniques are used in order to purify MBL. A study by Wang et al. [51] using chromatography observed that MBL was predominantly found in the chicken serum and was present in two forms with 26,500 and 75,000 Daltons. These forms represent the monomeric and trimeric form of MBL. 

### 5.2. Reverse Phase HPLC

Reverse phase high-pressure liquid chromatography (reverse phase HPLC) can purify MBL from other constituents of serum. In addition, it reveals the amount and concentration of MBL present in serum. However, this method is not suitable for molecules with higher affinity as its speed of detection reduces across the column. Laursen et al. [22] observed that MBL can be purified using reverse phase HPLC from other serum constituents. 

### 5.3. Enzyme-Linked Immunosorbent Assay (ELISA) 

This method is a specific serological test widely used for measuring the concentration of MBL in the chicken serum [48,49]. This method has been described as reliably producing consistent results. ELISA has the advantage that it does not require purified protein, since MBL can be measured directly in the blood [48,49]. However, ELISA reveals limited information about MBL. It mostly shows only presence and quantity of MBL but not the binding affinity. Several studies established that cMBL can be measured using such a so-called Sandwich ELISA [16,17,26,27,32]. In a study by Norup et al. [16], cMBL in serum of chicken was assessed using ELISA and showed a recognition limit of 0.6 mg/mL for MBL. In addition, ELISA also showed that MBL was detectable in the chicken embryo at 11 days before hatching (1.04 mg/mL) and increased to 5.0 mg/mL on hatching using ELISA method [16]. This concentration remains steady for 4 weeks and changes as age increases. Furthermore, it was observed that the MBL level in egg yolk and serum was equal. However, no MBL was found in the chicken albumen using this method. Overall, ELISA has proven to be a powerful and easy method to detect MBL levels in chickens.

### 5.4. DNA Typing 

DNA typing can be used for detection and precision testing for genetic variations of the MBL gene. This method aids prompt recognition of the presence of cMBL in the genome and, more specifically, genetic variations in the gene. DNA typing via PCR is based on amplification of the specific MBL gene, and is a highly reliable method which produces robust reproducible results [18]. However, the method depends on usage of appropriate or uncontaminated primers. Its sensitivity could lead to false results if the sample MBL protein is not well-handled. The method only provides information on the presence and exact sequence of the MBL gene but does not detect the actual gene expression levels. For this, quantitative (real-time) PCR is required. 

### 5.5. Real-Time PCR (qRT-PCR) 

Quantitative, real-time PCR (qRT-PCR) is broadly used for the detection and quantification of cMBL mRNA. This method requires fewer templates in comparison to the conventional PCR methods, and a main advantage is that it can be used in any tissue. The method can provide valuable information on basic levels of cMBL mRNA but is often used to detect up- or down-regulation of cMBL (or genes in general) upon infection [49]. However, it should always be kept in mind that mRNA levels do not necessarily correlate to protein levels of MBL, while the quantity of the actual MBL protein is what is functionally important. Kjærup et al. [41] observed a 3.4-fold rise in MBL gene expression derived from liver of L10H in comparison to L10L using real-time qRT-PCR [31]. This variation in serum concentration could be as a result of polymorphism in the promoter region of the MBL gene.

In another study by Ulrich-Lynge et al. [31], MBL gene expression was detected using qRT-PCR from chicken ceca. It was seen that L10H chickens have higher expression of MBL than L10L chicken on day1 post-infection (pi) and day 41 pi respectively [27]. Ulrich-Lynge et al. [27] performed an in vivo study to observe the cMBL and heterophil concentration using qRT-PCR. It was revealed that L10H chickens have as higher concentration of cMBL than L10L chicken on day 2 after inoculation (pi). Hence, it can be concluded that MBL is an acute-phase protein.

### 5.6. Mass Spectrometry 

Native mass spectrometry can be used to determine the total mass of a protein, but also its oligomerization state. However, one of its limitations is that its success depends on the purity of the sample and thus effective protein separation technology [49]. A study by Zhang et al. [19] used modified mass spectrometry in tandem with native mass spectrometry to detect the presence and oligomerization state of recombinant cMBL. Complex signals of three distributions of charged states were observed on individual species with different molecular weight of 26.1, 52.9 and 79.2 kilo Daltons, respectively [19]. From this data, it could be determined that recombinant cMBL was actually present mainly in its trimeric form with minute quantities of dimeric and monomeric species. Mass spectrometry detection of cMBL has not yet been done on native cMBL in more complex matrices, but with the fast recent developments in this technology, this should be feasible. 

## 6. MBL Localization/Synthesis

The cMBL and other members of the collectin family are all located at the end of the chromosome 6 [32] and the human collectin cluster is located on chromosome 10 [33]. Oka et al. [21] described MBL as the first C-type lectin member to be isolated among chicken species from the chicken liver. Several authors reported that cMBL synthesis is highest in the liver (hepatocytes) area while some lower quantities of MBL were seen in the larynx, infundibulum and abdominal sac [16,18,19,32]. Additionally, an infinitesimal amount of MBL mRNA was detected in the thymus, ovary and uterus region [32]. Likewise, Laursen and Nielsen [37] and Ulrich-Lynge et al. [18] detected the presence of cMBL in the germinal region of ceca, thymus, spleen and lungs of healthy chickens. In conclusion, cMBL can be seen in the reproductive, circulatory, digestive and respiratory system of chickens but at varying quantities.

## 7. MBL Concentration vs. Bacterial Disease Protection

As stated above, cMBL synthesis is known to occur mostly in the hepatocytes’ region, while limited quantities are found in the other organs. However, upon infection, higher MBL levels can be observed in specific organs due to local up-regulation. When there is an adequate quantity of MBL synthesized in the liver, the host organism will have the ability to combat bacterial pathogens at entry [17,18]. 

For example, Schou et al. [17] described that a low concentration of cMBL caused high disease prevalence in *Pasteurella multocida*-infected chickens, while Norup et al. [16] observed that high concentrations of MBL reduced chickens’ susceptibility to *Escherichia coli* [16]. Similar effects were observed for infections with *Salmonella enterica* and *Salmonella infantis* [42], while also, the outcome of viral infections seems to be related to serum levels of MBL [36]. Interestingly, there seems to be a strong genetic component that determines the concentration of MBL in chickens, with differences in average MBL levels between breeds [17]. Chicken strains specifically selected for high (L10H) and low (L10L) serum concentration of MBL have been bred and used to study the role of MBL in the chickens’ immune response. These studies consistently show that higher levels of MBL are correlated to lower bacterial disease in chicken. In one study, the amount of Salmonella shed in L10L chickens was higher than L10H chicken on day 4 pi. In chickens, it was shown that there was a higher level of Salmonella binding to MBL in chickens with high MBL levels (L10H strain) than in low-MBL chickens (L10L) at different days post infection (pi) [18]. This was suggested to be due to the huge role MBL plays in safeguarding chickens against Salmonella. Additionally, the L10L chicken excreted Salmonella for more days than the L10H chicken; this could be due to the presence of higher Salmonella counts in L10L chickens’ intestines than in L10H chickens. Schou et al. [17] also showed that chickens with low MBL concentrations were more prone to other pathogenic diseases. Different levels of binding of MBL to *S. aureus* NCT6571 strain (strong binding), *C. lusitanae* (strong binding) and *E. faecalis* (low binding) have been demonstrated [34] while Ulrich-Lynge et al. [18] and Seliger et al. [31] both observed that high cMBL levels and high monocyte numbers influence the clearance of *Escherichia coli* and some Salmonella spp of the C1 serotype. The lists of some of the reported cases of MBL affinity to bind bacterial diseases in chickens are presented in Table 3.

## 8. Antimicrobial Nature of MBL Concentration and Its Effect on Chicken Growth Rate 

The concentration of MBL has been known to be correlated to chickens’ wellbeing [14,16,26,52]. Since mannose-binding lectin has multiple antimicrobial activities, including (as stated above) aggregation of bacteria and increasing phagocytosis of bacteria, it is not easy to pinpoint exactly which of these activities has the largest positive effect on growth rate. However, higher levels of MBL likely protect better against pathogens, thereby diminishing the need for a more extensive (energy costly) immune response, which subsequently could lead to relatively more growth [24,30,31]. 

Several authors have reported variation in cMBL concentration from 0.4–35 µg/mL [23,32,35,41]. This variation was assumed to be caused by the single nucleotide polymorphisms (SNPs) in the different alleles present in exon 1 of the gene. In an in vivo study by Ulrich-Lynge et al. [18], where chickens were infected with *Salmonella infantis* (S.123443), an average MBL concentration of 21 µg/mL was detected in L10H chickens and 5.95 µg/mL for L10 L chickens [42]. The differences in MBL level were also associated to low body weight for L10 L chickens and they were also more susceptible to *Salmonella Montevideo* [18]. Finally, Norup et al. [16] also described that chickens with a high level of cMBL were less susceptible to *E. coli* and showed an increased body weight compared to chickens with low MBL (Table 2). Considering this correlation between MBL concentration and susceptibility towards bacterial infections, the concentration of MBL in chickens should be considered an important factor in future breed selection programs.

## 9. MBL Deficiency

Several studies have observed strong effects of MBL deficiency in humans [9,36,40,53]. MBL deficiency in humans is strongly related to severe bacterial infections in meningococcal meningitis and neutropenia patients among other infections [35], but little is known about complete MBL deficiency in chickens. Instead, lower levels of MBL have been described due to differences in the MBL promoter region. MBL deficiency or immunodeficiency can result in inadequate functioning of the IIS, providing an opportunity for pathogenic bacteria to infect [45,46,47]. A mutational effect of one amino acid in the exon 1 region of MBL can result in MBL structure disruption [9,16]. The disrupted region can cause a decline in MBL production [8]. 

The studies by Norup et al. [16], Ulrich-Lynge et al. [18] and Kjærup et al. [41] and on cMBL concentration observed an increase in bacterial infection as cMBL production declines. Therefore, to ensure adequate clearance of bacterial infection in chickens, there is need to select chickens with normal or high MBL concentration as cMBL insufficiency has a deleterious effect on chicken and human health. 

## 10. Possible Factors Affecting cMBL Production in Chicken Management Systems

Many factors are responsible for the production of cMBL thereby affecting the health of chickens in general. This section seeks to discuss the effect of some of the factors on cMBL secretion in chicken production and provides a possible approach to facilitate cMBL secretion in chickens.

### 10.1. Effect of Age and Stress on cMBL Production

Age of chicken is one of the factors that can influence the level of cMBL production in chickens. Nielsen et al. [15], Norup et al. [16] and Schou et al. [17] discovered that cMBL quantities remain stable from day-old chicks until 5–7 weeks of age and also remain stable from 9 to 18 weeks of age and 19 to 38 weeks of age, irrespective of the sex of the chicken.

However, in the event of inoculating chickens with bacteria, an increase in basal MBL protein levels at 19 weeks was detected when challenged with *E. coli* O78 K80, after which the amount of MBL remained stable [16]. No effect of sex on MBL levels was observed in this study, since both male and female chickens had the same MBL levels. Laursen and Nielsen [23] showed a similar but even higher (100%) up-regulated MBL level. The difference may have been due to chickens being co-exposed to a viral infection in the latter study. 

Exposure to stress could also affect the production of MBL in chickens. In unpublished work, stress was stated to increase the MBL level in chickens, according to Schou et al. [17]. Similarly, a study by Norup et al. [54] observed that when chickens are exposed to a non-infectious stressful situation, the cMBL level increases. The level of stress induction of cMBL is dependent on chicken breeds and, obviously, the degree of stress. More studies should be conducted to establish which different stressors can affect cMBL production.

### 10.2. Effect of Breed on cMBL Production

The effect of breed on cMBL production has been poorly studied as there are many other factors in relation to breed which cause variation in cMBL production. 

An inter-breed effect on MBL was observed between ISA Brown and the Hellevad breed; the mean serum MBL concentrations were 6.57 and 18.81 mg/mL respectively [26]. This indicates that MBL levels are influenced with breed variation. However, Schou et al. [17] revealed that differences in the cMBL concentration can vary between individual chickens within the same breed even when there was no statistical difference between the two breeds studied (Ri 539 and Luong Phuong chickens). It was concluded that the MBL allele can vary in the population since chickens are from different maternal lineage, even when chickens are of the same breed. Interestingly, in this study, a significant difference in resistance to *Pasteurella multocida* infection between the two chicken breeds was observed. This could possibly be related to a genetic potential for higher humoral response in Ri chicken than in Luong Phuong chickens, but it could very well also be related to intra-breed differences in MBL concentration with infection only documented in Ri birds with low serum cMBL. In another study, Nielsen et al. [15] stated that egg-laying breeds tend to be more prone to infection than meat-type breeds due to differences in cMBL concentration. The same study observed an increase in the mortality rate of egg-laying breeds compared to meat-type breeds. 

From the studies above, it can be concluded that conscious selection of chickens with a high/moderate MBL plasma concentration is vital as the young chicken’s immune system has not been fully developed. Therefore, in order to maintain chicken breeds resistant to diseases, genetic selection of high MBL breeds may be of great value to successful chicken production. 

### 10.3. Possible Effect of Feed Manipulation on cMBL Expression 

Recent chicken feeding strategies aim to optimize the potential of the innate immune response in chickens. This plan is adopted to strengthen the health status of chickens and to increase productivity [55]. Several studies have shown that probiotics enhanced the response of the IIS [56]. In fact, Pascal et al. [57] reviewed that probiotics can directly inhibit the development of microbes via production of antibacterial substances such as bacteriocins, and some kinds of acid: propionic, acetic and lactic acids. Probiotics can also limit the adhesive capacity of pathogens to the gastrointestinal tract of chickens by sticking to the epithelial cells. Nevertheless, to date, there have been few research studies on the modulating ability of probiotics on MBL expression in chickens, however, it is an interesting hypothesis that should be investigated. 

Some studies have either supplement fed and/or orally challenged chickens with mannose, mannan oligosaccharides, yeast protein concentrate, pellet of yeast protein concentrate and even whole yeast and *Saccharomyces boulardii*. These studies showed greatly reduced bacterial colonization such as *Salmonella typhimurium* from day 7 to day 14 pi in the chicken intestine [58,59,60,61]. It can be deduced that binding needs attachment of bacterial lectin to receptors having mannose at the cell wall and presence of mannose can effectively block bacterial lectins on the yeast cell wall. 

It can also be suggested that feeds formulated with sufficient calcium supplements could enhance the production of cMBL in chickens. This was due to MBL’s nature to bind calcium ions and its calcium dependent (Ca^2+^) activity. Furthermore, inclusion of essential oil (components), such as cinnamicaldehyde and thymol blended in a wheat-based diet has been known to reduce the microbial count of Salmonella and *E.coli* in the chicken fecal sample and to improve the function of the IIS; there could be a link between essential oil inclusions on cMBL production [62,63] but this has not been completely established. However, this attempt to establish a link between dietary manipulations and cMBL secretion will be potentially an important tool to influence MBL production in chickens. 

## 11. Conclusions

This review describes that the presence of MBL influences the innate immune response in combating pathogenic bacterial diseases. MBL can be detected and quantified by various methods. MBL deficiency could be a threat to chickens and this can lead to high susceptibility and, in extreme cases, mortality. This study reveals many factors that affect cMBL production such as age, stress, breed and possibly, feed manipulation strategies. Hence, there is a need to consciously select chicken breeds with adequate quantity of cMBL in order to reduce the arbitrary use of antibiotics. In conclusion, the knowledge of MBL and its effects on the resistance of chickens to bacteria can be explored as an excellent tool for disease control in chicken production. Yet, there is need to explore the potential SNPs responsible for low cMBL production in some chicken breeds and the direct impact of feed manipulation on cMBL production as this can help to develop a more profitable breeding program.

## Figures and Tables

**Table 1:** Features of different detecting techniques of MBL in chicken.

Identification Method	Mode of Action and Sensitivity	Price and Sample Size	References
Affinity chromatography	Less sensitive Possibility of interfering with MBL structure, ligand leakage.	Small sample ranging from 1–10 samples, consumes time, less reliable and less reproducible	[22,48]
Enzyme-linked immunosorbent assay (ELISA)	Specific monoclonal and polyclonal detection Very sensitive and reliable	Expensive and medium sample size	[23]
Polymerase chain reaction (PCR) Real time PCR Multiplex PCR	Use of suitable primers Flourescent reporter signal Sequence specific probe Proper genotyping of MBL gene	Extremely expensive, highly reproducible and Highly reliable with large sample size.	[49]
Mass spectrophotometry	mass of heterogeneously glycosylate protein; molecular weight of MBL	Highly reproducible with high sample size	[19]

**Table 2:** The impact of cMBL on bacterial disease and growth rate in chicken.

Treatment	Age of Chicken	Site of MBL	Detection Method	Outcome/Result	References
Consequence of low mannose-binding lectin plasma concentration in relation to susceptibility to *Salmonella infantis* in chickens	Day-old	Serum and Ceca	ELISA, RT-PCR and Flow Cytometry	Higher average body weight gain and MBL expression in L10H. Higher bacteria count in caecum swab of L10L	[42]
*Escherichia coli* ISA, LSL, LB, HE	16–52 weeks old	Serum	ELISA and PCR	Increased body weight in high MBL. Low MBL chicken are more prone to *E. coli*	[16]
Effect of *Pasteurella multocida* inoculation on MBL level	16 weeks	Serum	ELISA	Significant low MBL in systemic infected chicken with *Pasteurella multicoda* (acute phase)	[17]
Broilers with low serum mannose-binding lectin show increased fecal shedding of *Salmonella enterica* serovar Montevideo	Day old	Serum	ELISA and RT-PCR	L/H chickens had significantly less salmonella counts/cloacal swab at week 3and 5 post infection than L/L chicken. Chicken with low MBL are more susceptible to Salmonella thanhigh MBL chicken.	[50]
Chicken mannose-binding lectin function in relation to antibacterial activity towards *Salmonella enterica*	NA	Serum	ELISA and Flow-Cytometry	*S. enterica S. enterica* is the only C1 serotypes that bound to cMBL.Serum with high cMBL exhibited a greater bactericidal activity to *S. aureus* than serum with low concentrations of cMBL activity to *S. aureus* than serum with low concentrations of cMBL	[18]

**Table 3:** Selected reports of cMBL (chicken mannose-binding lectin) bacterial binding.

Bacteria Strain	Site of Binding	References
*Pasteurella multocida*	Liver, spleen, serum	[17]
*Escherichia coli*	Liver, serum	[16,32,34]
*Salmonella enterica*	Liver, serum	[18]
*Staphylococcus aureus*	Ceca, serum	[33,35]
*Klebsiella oxytoca*	Serum	[34]
*Klebsiella pneumoniae*	Serum	[34]
*Pseudomonas aeruginosa*	Serum	[34]
*Yersinia pseudotuberculosis*	Serum	[34]
*Salmonella Typhimurium*	Serum	[19]
*Salmonella Montevideo*	Serum	[41]
*Enterobacter cloacae*	Serum	[34]

## Data Availability

No new data were created or analyzed in this study. Data sharing is not applicable to this article.
